# Effects of cell morphology, physiology, biochemistry and *CHS* genes on four flower colors of *Impatiens uliginosa*

**DOI:** 10.3389/fpls.2024.1343830

**Published:** 2024-03-01

**Authors:** Lu-Qiu Zhao, Yang Liu, Qi Huang, Shan Gao, Mei-Juan Huang, Hai-quan Huang

**Affiliations:** College of Landscape Architecture and Horticulture Sciences, Southwest Research Center for Engineering Technology of Landscape Architecture, Yunnan Engineering Research Center for Functional Flower Resources and Industrialization, Research and Development Center of Landscape Plants and Horticulture Flowers, Southwest Forestry University, Kunming, China

**Keywords:** flower color, anthocyanins, cellular pH, *CHS* genes, *Impatiens uliginosa*

## Abstract

**Introduction:**

Flower color is one of the important ornamental traits in the plants, which plays an active role in attracting pollinators to pollinate plants and reproduce their offspring. The flower color of *Impatiens uliginosa* is rich, there are four main flower colors in nature: deep red, red, pink, and white. However, it remains unclear whether on four different flower colors mechanism of *I. uliginosa*.

**Methods:**

We investigate colorimetric measurement, observation of epidermal cells, cellular pH determination, extraction and determination of total anthocyanins and flavonoid, semi-quantitative determination of pigment components, and gene cloning and qRT-PCR of *CHS* genes to study four flower colors of *I. uliginosa*.

**Results:**

The L^*^ and b^*^ values were the highest in white flower, while the a^*^ values were the highest in pink flower. The same shape of epidermal cells was observed in different flower colors, which was all irregular flat polygons, and there were partial lignification. Their cellular pH values were weakly acidic, while the pH values of the deep red flower was the highest and the white flower was the lowest. The highest pigment content of the four flower colors was total anthocyanin content. And malvidin-3-galactosidechloride (C_23_H_25_ClO_12_), cyanidin-3-O-glucoside (C_21_H_21_O_11_) and delphinidin (C_15_H_11_O_7_) were the main pigment components affecting the color of four different flower colors. The anthocyanin synthesis gene *IuCHS* was expressed in four flowers, and all three copies of it had the highest expression level in pink flower and the lowest expression level in white flower.

**Discussion:**

These results revealed the influence of main internal factors on four different flower colors of *I. uliginosa*, and provided a basis for further understanding of the intracellular and molecular regulatory mechanisms of flower color variation, and laid a foundation for the improvement of flower color breeding of *Impatiens*.

## 1 Introduction

Different flower colors can not only attract pollination by insects, which is conducive to plant reproduction, but also increase the ornamental value of plants (Riffell et al., 2013). In addition to external environmental factors (Ben-Tal and King, 1997), internal factors such as flower pigment types and contents, epidermal cell shapes, cellular pH values and regulation of anthocyanin synthesis genes also play important roles in the formation of flower color.

Flower color is mainly determined by carotenoids, flavonoids and other compounds (Forkmann, 1991). Carotenoids are important compounds in the plant photosynthetic system, which can give flowers and fruits different colors from yellow to red (Volker, 2019). Flavonoids are secondary metabolites of phenylpropane metabolic pathway, which make flowers and fruits of plants appear light yellow to blue (Chen et al., 2013). The study on the flower color of *Impatiens balsamina* mainly focuses on anthocyanins (Ralph and Charles, 1958; Charles, 1959; Aminah et al., 2019), which are water-soluble pigments of flavonoids and show different colors depending on the acidity and alkalinity of the cells fluid. Anthocyanins widely distributed in the plants are centaurin, pelargonidin, delphinidin, paeoniflorin and so on (Holton and Cormish,1995; Harborne and Williams, 2000).

Anthocyanins are generally found in upper epidermal cells, but they are also present in palisade tissue and sponge tissue cells of darker petals (Gonnet, 2003; Kugler et al., 2007). It has been found that the formation of purple flowers of calamus is not only related to the content of anthocyanin, but also influenced by the length and arrangement order of the perianth epidermal cells (Yoshida et al., 2003). In addition, cellular pH regulates petal color by changing anthocyanin conformation and absorption spectrum (Pina, 2014). Anthocyanin is red and stable at the low pH value, and flower color tends to be blue and unstable with the increase of pH value (Tanaka et al., 1998).

Genes play an important role in anthocyanin biosynthesis. The expression of these genes is closely related to anthocyanin content, thus affecting the flower color of plants (Morita and Hoshino, 2018). Anthocyanin biosynthesis genes, such as chalcone synthetase (*CHS*), chalcone flavanones (*CHI)*and flavonoid 3-hydroxylase (*F3H*), have been well identified. *CHS* gene is the first cloned flavonoid secondary metabolic synthetase gene and is first identified in *Petroselinum crispum* (Reimold et al., 1983). It is a polygenic family with different number of family members in different plants, and the expression patterns of different members are also different (Han et al., 2006). In many species, multigene families of *CHS* have been identified. For example, 14 complete *CHS* genes have been identified in maize (Han et al., 2016). In *Phalaenopsis*, three *CHS* genes are isolated, and their expression patterns in floral tissues at different developmental stages are diverse (Han et al., 2017). Of the three *CHS* genes, *PhCHS5* is most highly expressed and is the sole *CHS* gene responsible for pigment accumulation.

*Impatiens uliginosa* is a herbaceous plant of the genus *Impatiens* in the Impatiens family, mainly distributed in Yunnan, Guizhou and Guangxi, China. It is an important ornamental plants that exhibits great flower color diversity. There are four main flower colors in nature: deep red, red, pink, and white, in which red is the wild type and the other three are the mutant type. Compared to a single flower color, different flower colors of *Impatiens uliginosa* can attract people to admire. However, few people have studied the different flower colors of *I. uliginosa*. Studies have shown that flowers color is mainly due to the presence of pigments (Kanehisa et al., 2008; Zhao et al., 2011; Van Der Kooi et al., 2019), and the study of impatiens color mainly focused on the anthocyanins. Therefore, we wanted to explore whether anthocyanins are the main factors affecting the formation of the four different flower colors of *I. uliginosa*, and whether there are other influencing factors besides anthocyanins. In this study, the mechanism of color and color variation of four different flower colors (deep red, red, pink and white) was studied from the aspects of phenotype, morphological anatomy, pigment types and contents, and anthocyanin synthesis gene. Understanding the regulatory mechanisms of these factors in the coloration of *I. uliginosa* will have great implications for the breeding of flower color in *Impatiens*.

## 2 Materials and methods

### 2.1 Material and collection place

**Figure 1:**
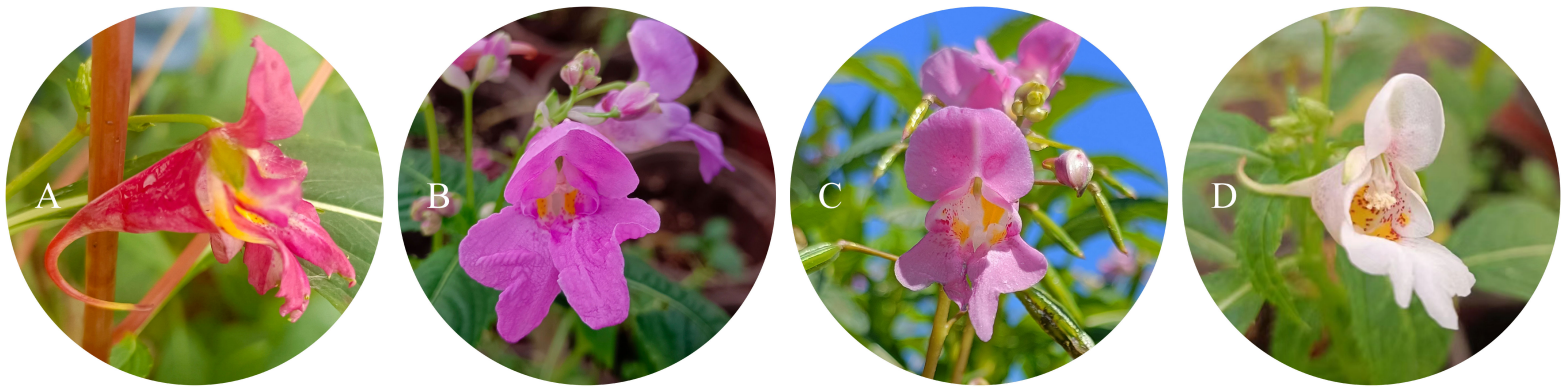
Four different flower colors of *I. uliginosa.* **(A)** Deep Red (DR); **(B)** Red (R); **(C)** Pink (P); **(D)** White (W).

The experimental materials were four different flower colors of *I. uliginosa* collected from Aziying, Laoyu River and Anning Dadie water in Kunming (**Figure 1**). The detailed information of collection sites was shown in **Supplementary Table S1**.

### 2.2 Colorimetric measurement

Fresh petals were collected and measured under the conditions of CIE10°standard color function and CIE standard D65 light source, and the average values of three measurements were used. After the measurement, the chromatic value of flower colors were analyzed according to the CIE L^*^a^*^b color model of the International Commission on Illumination (CIE) (Gonnet, 1998).

### 2.3 Observation of epidermal cells

The fresh petals were stored in FAA fixing solution (50% ethanol 90 mL + glacial acetic acid 5 mL + 37% formaldehyde 5 mL), rinsed with tap water and sliced by conventional paraffin wax method (Li et al., 2022). The slices were sliced by a slicer (LEICA RM205), the thickness was 8 μm, and the slices were stained with saffred solid green, observed and photographed with an electron microscope (LEICA DMi 1).

### 2.4 Cellular pH determination

Fresh and intact petals picked, quickly grind them into a centrifuge tube and centrifuge for 4min (5°C, 12000 rpm), and the cellular pH values of petal was measured with the supernatant using a pH meter (Reuveni et al., 2001). Each sample was measured three times and averaged.

### 2.5 Extraction and determination of total anthocyanins

The total anthocyanins (TA) content of four different flower colors was measured according to the protocol within Equation 1 (Fuleki, 1969). The dried petal powder was 0.3 g and crushed to obtain three replicates, and soaked in 1% hydrochloric acid methanol solution for 24 hours at a constant volume of 25 mL. The UV-visible spectrophotometer was set at 200 V and scanned at a wavelength of 520-550 nm. The total anthocyanin contents were calculated using the following equation (Zhang and Shen, 2014):


(1) Anthocyanin content(ug/g)=(D∗V∗n∗10)/(98.2∗m)

where D is the absorbance of the pigment at the maximum absorption wavelength. V is the constant volume (mL) when extracting pigment from a certain mass of petals; n is the dilution multiple during colorimetry; 98.2 is the average extinction coefficient of anthocyanin pigments at 535nm wavelength, and m is the usage of samples (g).

### 2.6 Extraction and determination of total flavonoids

Took 0.1 g of dried petal powder and added it to 10 mL of extraction agent methanol for 48 hours, concentrated it under vacuum after filtered it, and filled it with methanol solution to 25 mL. Then added 25 mL of pure water and shook it evenly, and extracted it with 50 mL of petroleum ether, shook vigorously, and let stand for 30 minutes. Discarded the upper layer of petroleum ether and repeat the extraction three times before collecting the aqueous phase. The aqueous part was added to ethyl acetate for three times to obtain the extraction solution, and the methanol solution was added to 25 mL to obtain the extraction solution. Prepared 0.77 mg/L rutin standard solution, added 6 mL of 1% AlCl_3_·6H_2_O methanol solution, and plot the standard curve with absorbance at 415 nm (D270 nm). Took 2 mL of extraction solution, added 3 mL of AlCl_3_·6H_2_O methanol solution, and obtained the total flavonoid (TF) content of four different flower colors with absorbance at 415 nm (Shraim et al., 2021).

### 2.7 Semi-quantitative determination of pigment components

Extracted 0.5 g of each petal, extracted 3 parts in total, and soaked it in 150 mL 0.1% hydrochloric acid methanol solution for a week. After filtration, vacuum concentration was obtained to obtain anthocyanins. The filtration with microporous filter membrane, and the anthocyanins were stored in a fixed volume of 25 mL for use. The HPLC instrument (Ulitimate 3000 LTQ ORBITRAP XL) was equipped with column C18 (100 mm*2.1mm), and the eluent was methanol: water (0.1% containing formic acid) =98:2, and the flow rate was set at 0.3mL/min. The total relative anthocyanin content of each sample was based on the total area of all HPLC peaks relative to each other in the chromatogram. Possible pigment components in petals were searched (**Supplementary Table S2**), and the peak area of pigment was integrated after retrieval.

### 2.8 Cloning and quantitative reverse transcription-polymerase chain reaction of *IuCHS* gene

By using RNA extraction kits (Plant RNA kit, Omega, Norcross, Georgia) to extract total RNA from four different colors. Reverse transcription was using cDNA Synthesis SuperMix (TransGen, Beijing, China). The *IuCHS* gene was cloned by combining the transcriptome data and RACE (rapid-amplification of cDNA ends) technique, and the primers used for cloning were listed in **Supplementary Table S3**. Alignment of the sequences amino acids of *IuCHS* was carried out using the ClustalW algorithm in DANMAN software (LynnonBiosoft, USA) (Zhang et al., 2024), and a phylogenetic tree was constructed with the CHS protein sequences of the other species and IuCHS using the neighbor-joining method in MEGA11 software (Tamura et al., 2021), with 1000 bootstrap replicates and the other parameters set to default (Nei and Kumar, 2000). The relative expression of *IuCHS* gene was calculated using 2^−ΔΔCt^ method (Sun et al., 2017). Primers for quantitative reverse transcription-polymerase chain reaction (qRT-PCR) were listed in **Supplemental Tables** (**Supplementary Table S4**).

### 2.9 Statistical analysis

All assays were performed in triplicate, and data were expressed as the mean ± standard deviation. Using repeated measures and one-way analysis of variance (ANOVA) process of the general linear model in SPSS and giving comparison among different groups pairwise (Armstrong and Everett, 1990). Duncan’s multiple range test was used to determine that the difference was statistically significant (p<0.05). Poundwise comparison was conducted among different groups, and Bonferroni value was used as the final result.

## 3 Result

### 3.1 Measurement of colorimetric values

**Figure 2:**
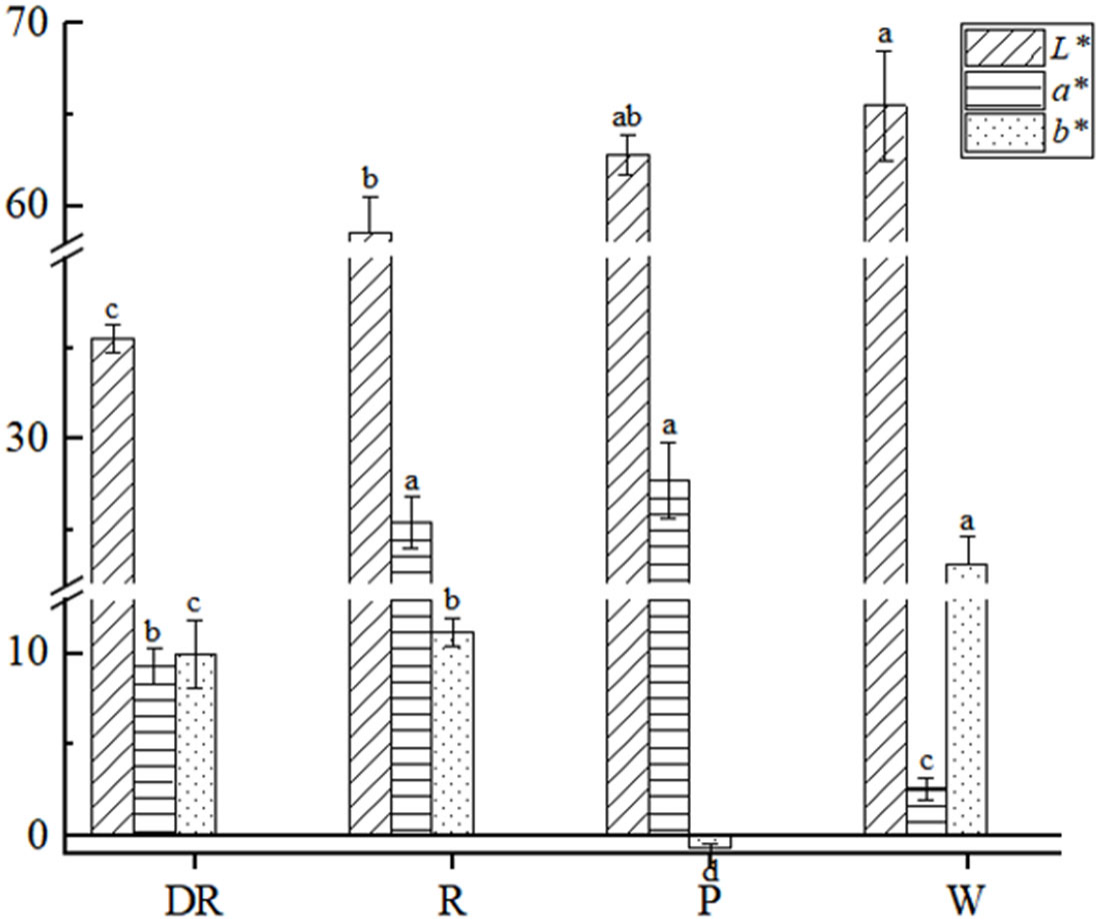
Colorimetric values of four different flower colors of *I. uliginosa.* Data are shown as the mean ± standard error of the mean, based on three replicates. Different alphabets indicate significant differences at p<0.05 according to the Duncan’s multiple range test.

The L^*^ value of four different flower colors ranged from 35.5 to 65.5, in which L^*^ value of the deep red flower was the lowest, and the white flower was the highest. The L^*^ value showed a negative correlation with flower colors. The deeper the color of flowers, the lower the L^*^ value. The a^*^ value of the white flower was the lowest, and the pink flower was the highest. The b^*^ value of the four flower colors was relatively variable, ranging from -0.7 to 23.2, where b^*^ value of the white flower was the highest and the pink flower was the lowest (**Figure 2**). Bivariate correlation analysis of L^*^, a^*^ and b^*^ values was performed by SPSS, there was no significant correlation (p<0.05) between them (**Supplementary Table S5**).

### 3.2 Morphological analysis of petals epidermal cells

**Figure 3:**
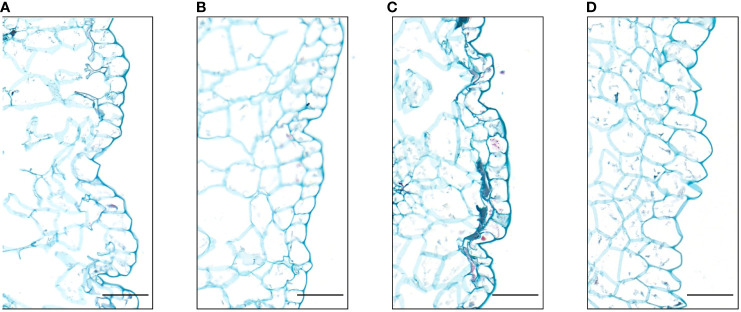
Paraffin sections of the epidermis cells of the petals of *I. uliginosa *(100×). **(A)** Deep Red (DR); **(B)** Red (R); **(C)** Pink (P); **(D)** White (W). Bar=100μm.

We observed the petals epidermal cells of the four different flower colors. The results showed that the shape of their epidermal cells were mostly irregular flat polygons, and there were partial lignification. However, some differences were observed in cell size among these four flowers. The white flower had a larger epidermal cell than the other three flowers, whereas the cell size of three flowers was no significant difference (**Figure 3**). Thus, the shape and size of epidermal cells did not affect the color of the four flower in *I. uliginosa*.

### 3.3 Cellular pH determination of petal

**Figure 4:**
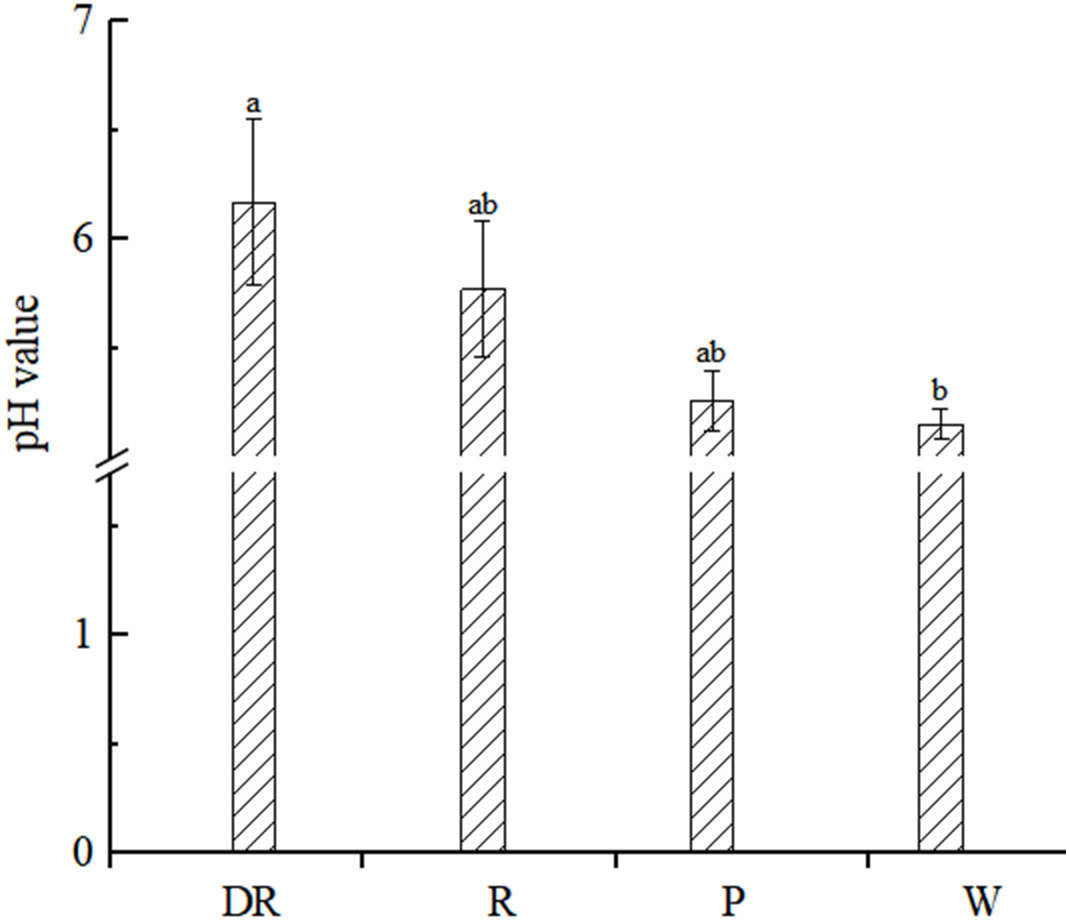
Petal vacuolar pH value. Data are shown as the mean ± standard error of the mean, based on three replicates. Different alphabets indicate significant differences at p<0.05 according to the Duncan’s multiple range test.

The cellular pH values of the four flower colors were weakly acidic, and the pH values of the four flower colors were deep red > red > pink > white (**Figure 4**). The result showed that the pH values of the flower colors were positively correlated with the depth of flower colors. And the correlation analysis of pH value and L*a*b* value showed that they had no significant correlation (p<0.05) in four flower colors (**Supplementary Table S6**).

### 3.4 Determination of the total anthocyanins content and the total flavonoids

**Figure 5:**
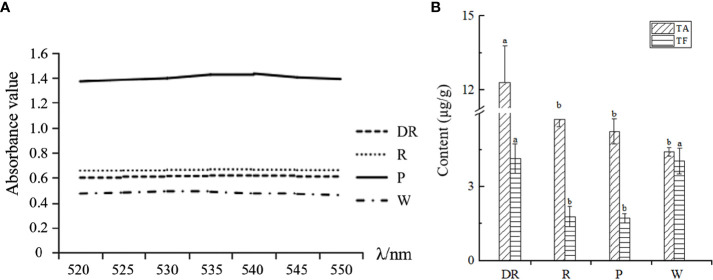
**(A)** Wavelength scanning results for the determination of total anthocyanins. **(B) **The total anthocyanin content (TA) and total flavonoid content (TF) of four different flower colors of *I. uliginosa*.

Wavelength scanning was performed at 520-550nm for four kinds of anthocyanin solutions of different flower colors, and the absorption value of anthocyanin solution was determined after the maximum absorption wavelength was obtained. We found that the maximum absorption wavelength of the deep red flower was 540nm, whereas the maximum absorption wavelength of the other three flowers was 535nm (**Figure 5A**). Under the maximum absorption wavelength, the anthocyanin content of four flower colors was determined. The anthocyanin content in deep red flower was the highest and lowest in white flower, and the anthocyanin content was positively correlated with the depth of flower colors. To conclude, the difference in anthocyanin content may be the reason for the color difference of the four flower colors.

According to the standard curve of flavonoids (**Supplementary Table S7**), the content of total flavonoids of four flower colors was obtained (**Figure 5B**). The content of total flavonoids in deep red flower was the highest and most lowest in red flower. Our results showed that the contents of total anthocyanins and total flavonoids in deep red flower were the highest. However, the anthocyanin content of the other three flowers was inversely proportional to the flavonoid content.

### 3.5 Semi-quantitative determination of pigment components

**Figure 6:**
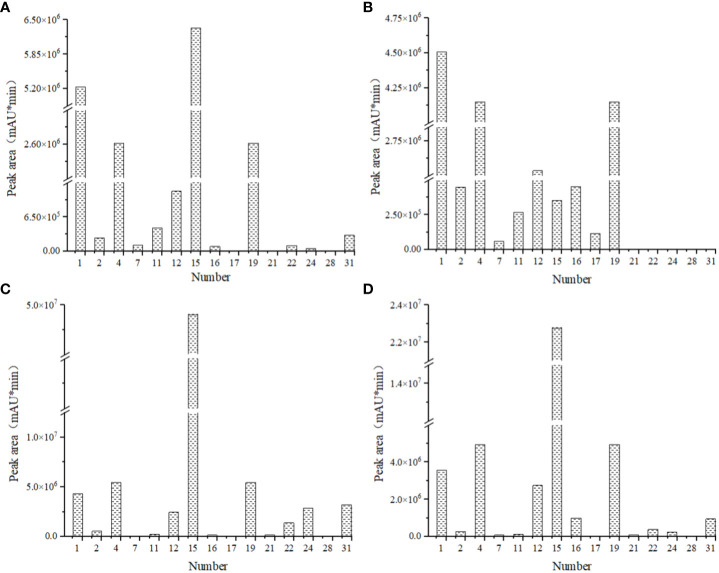
Comparison of pigment species and contents of four kinds of flowers in *I. uliginosa*. **(A) **Deep Red (DR); **(B) ** Red (R); **(C) **Pink (P); **(D)** White (W). 1, cyanidin-3-O-glucoside; 2, cyanidin 3,5-diglucoside chloride; 4, delphinidin; 7, malvidin chloride; 11, petunidin chloride; 12, malvidin-3-Oarabinoside chloride; 15, malvidin-3-galactoside chloride; 16, kaempferol-3-O-rhamnoside; 17, kaempferol-4’-glucoside; 19, quercetin; 21, myricetin; 22, jaceosidin; 24, peonidin-3-O-galactoside; 28, dihydroquercetin; 31, myricitrin. The sample volume, extraction liquid volume and extraction time of *I. uliginosa* were the same, so the relative pigment content of *I. uliginosa* could be compared by chromatographic peak area (mAU*min).

Among the 35 pigments retrieved, there were 9 pigments were found in four flower colors, and 6 pigments were found only in some flower colors (**Figure 6**). The red flowers contained the fewest anthocyanin species, while pink flowers contained the most. The content of cyanidin-3-O-glucoside in red flowers was the highest, while the content of malvidin-3-galactoside chloride in the other three flowers was the highest, this may be the main reason for the difference between pink flowers and the other three flower colors. The lowest content of peonidin-3-O-galactoside was found in deep red flowers and the lowest content of dihydroquercetin was found in pink flowers. Both red and white flowers had the lowest malvidin chloride content. To conclude, the types and contents of anthocyanins in the four flower colors were different, so it was speculated that the different kinds and contents of anthocyanins may be the reason for the difference of the four flower colors. In other words, the formation of flower colors was not determined by a single anthocyanin species.

### 3.6 The cloning and expression of *CHS* gene in four different flower colors of *I. uliginosa*

**Figure 7:**
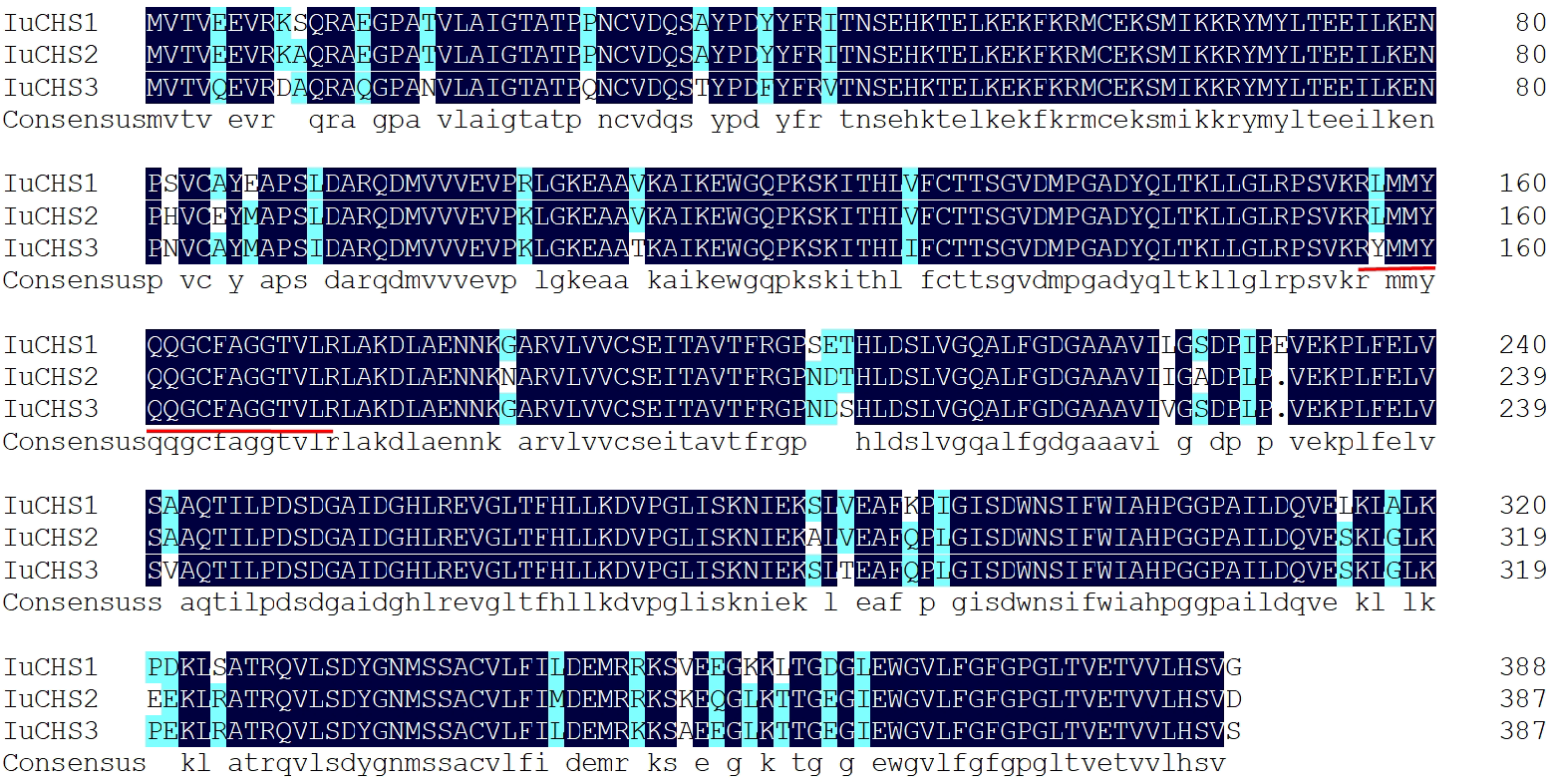
Alignment amino acid sequences of CHS genes of *I. uliginosa.* The characteristic protein sequences of the Chalcone synthase superfamily are underlined in red.

**Figure 8:**
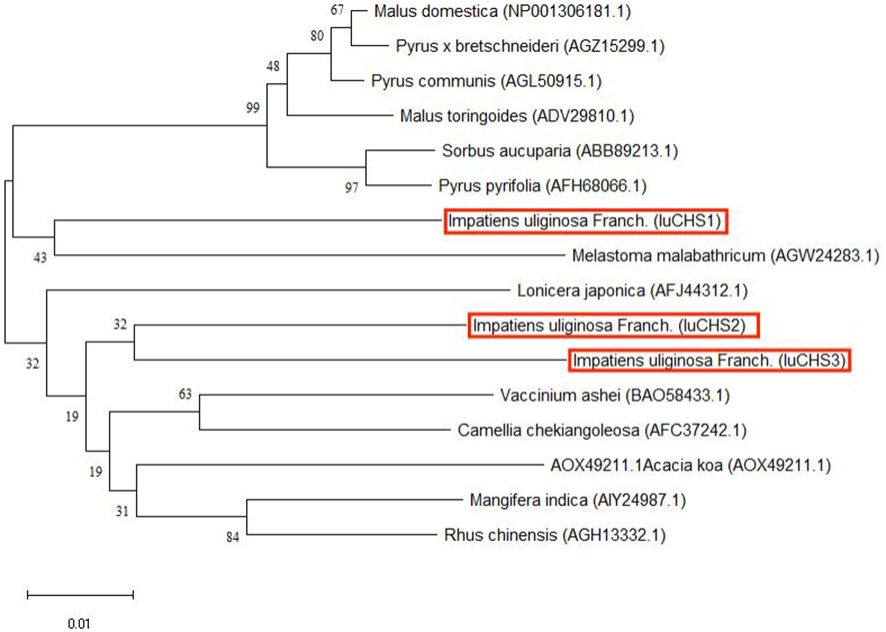
Phylogenetic tree of *CHS* genes of *I. uliginosa.* The phylogenetic tree analysis of the full-length sequences of CHS proteins was constructed using the Neighbor-Joining (NJ) method in MEGA 11. The *CHS* genes of *I. uliginosa* is marked in red.

According to the transcriptome data of our research group, there were three copies of *CHS* gene in the four flower colors, and the sequence was consistent. Three copies of the *CHS* gene were successfully cloned in *I. uliginosa*, named *CHS1*, *CHS2* and *CHS3* respectively, with a total cDNA sequence length of 1170 bp, 1167 bp and 1167 bp, and encoded 388, 387 and 387 amino acids respectively (**Supplementary Figure S1**). The amino acid sequence identity of the three *CHS* genes was as high as 95.12%, and all of them contained the characteristic protein sequences of the Chalcone synthase superfamily (**Figure 7**). The phylogenetic tree was constructed according to the amino acid sequence of CHS and the best model LG+G was selected, and the results of constructing their phylogenetic tree (NJ) indicated that the *CHS1* gene was clustered with *Melastoma malabathricum* together, while the *CHS2* and *CHS3* genes were clustered together (**Figure 8**). Therefore, the *CHS2* and *CHS3* genes were more closely related than the *CHS1* gene in *I. uliginosa*.

**Figure 9:**
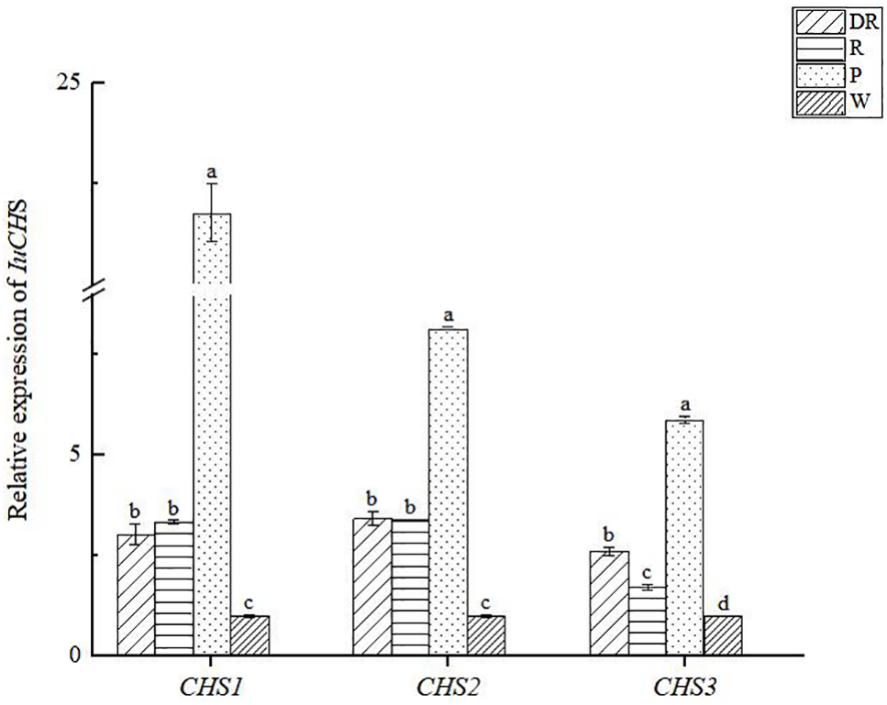
The relative expression of *CHS* genes in four flower colors of *I. uliginosa*. Data are shown as the mean ± standard error of the mean, based on three replicates. Different alphabets indicate significant differences at p<0.05 according to the Duncan’s multiple range test.

To investigate the expression profile of *CHS* genes in full-bloom stage were determined by qRT-PCR (**Figure 9**), and the variance analysis of CHS genes (**Supplementary Tables S8–S11**). The results showed that the three *CHS* genes were expressed in four flower colors. Comparatively, the expression level of *CHS* genes was highest in pink flower, with *CHS1* gene being particularly high, whereas lowest in white flower. In addition, we also found that the expression levels of the three *CHS* genes differed significantly in pink flowers, while the differences were not significant in the other three flower colors. In conclusion, *CHS* genes had certain regulation effect on the four flower colors formation of *I. uliginosa*, which may play a more important role in the formation of pink flower.

## 4 Discussion

The formation mechanism of flower color in plants has been the research subject of many scholars. Many environmental factors affect the color of flowers (Dela et al., 2003). For example, temperature exerts obvious effects on petal color by causing changes in the anthocyanins content (Stiles et al., 2007; Lai et al., 2011). Cultivation management, such as watering, can also affect the floral color to some extent (Aminah et al., 2019). In addition to this, other internal factors also play an important role in the formation of color. *Impatiens balsamina* flower has a variety of colors and contains anthocyanin pigments (Zhang et al., 2018). This study evaluated the effects of the phenotype, cellular pH, anthocyanin species and content, and anthocyanin synthesis genes of four different flower colors in *I. uliginosa*.

The stimulation of color in human eyes can’t be represented by quantitative data, so people often use CIE color measurement system to realize the data of color phenotype (Lu et al., 2021). It is found that.L^*^ and a^*^ in flowers were closely correlated (Lei et al., 2017). In the studies of *Paeonia lactiflora* (Wang et al., 2013) and *Dendrobium phalaenopsis* (Li et al., 2013), there is no correlation between L^*^a^*^b^*^. In this study, it was found that L^*^ value of the deep red was the smallest, L^*^b^*^ value of the white was the largest and the a^*^ value was the smallest. In addition, a^*^ value of the pink was the largest and the b^*^ value was the smallest. There was no correlation between the L^*^a^*^b^*^ values. It was speculated that the phenotypic parameters of different plants may not necessarily be correlated due to the differences between them.

The color of *Antirrhinum majus* mixta mutant changes from purple to pink, and the shape of its epidermal cells changed from conical to flat (Whitney and Glover, 2007). However, the color of some plants is independent of the shape of epidermal cells. For example, the epidermal cell structure of two flowers of different colors on the *Hyacinthus orientalis* inflorescence is the same (Qi et al., 2013). In this study, the four different flower colors of the epidermal cells were oval, indicating that the flower color of *I. uliginosa* was not related to the structure of the epidermal cells. Studies have been shown that the color of anthocyanins was pH-dependent, the pH value increased from 5.5 to 6.6, and the color of many cells changed from reddish purple to purplish blue (Asen et al., 1975). And anthocyanins accounted for coloration of ray florets in red and pink chrysanthemums, and the cytoplasmic pH value was positively correlated with the color depth (Wang et al., 2022). In this study, the vacuolar pH values of four different flower colors were all between 5 and 7, the vacuolar pH values of white petals were 5.15, and the vacuolar pH values of deep red petals were 6.17, indicated that the deeper the flower color was, the higher the vacuolar pH values were.

Flower color of plants is the result of the synergistic action of many factors, but the spatiotemporal combination of different types and contents of pigments in petal cells ultimately determined the flower color, and the color is not exactly the same as the color of pigments contained in petals (Kishimoto and Ohmiya, 2006; Alquezar et al., 2009; Huang et al., 2009). Flavonoids and anthocyanins are readily soluble in water and are mainly find in the vacuoles of plant cells, produce the full range of colors from deep red to reddish purple (Hirschberg et al., 1997). Our result was found that the anthocyanin content and flavonoid content were the highest in the deep red, and the color depth of different colors was positively correlated with the content of anthocyanin, so anthocyanin may be the main color pigment of *I.uliginosa*. And the difference of the four flower colors should not be caused by a certain anthocyanin, but by the combination of multiple anthocyanins, which ultimately caused the color difference of the petals we saw. Meanwhile, it was also found that the pink flowers contained some unique pigments. Whether these pigments were responsible for the formation of the pink flowers was also unknown and needed to be proved through later tests.

The *CHS* genes is widely find in various plants and plays an important role in plant physiology and biochemistry (Ma et al., 2016). They are expressed in floral tissues and involved in anthocyanin synthesis, that have different expression levels and expression patterns in the plants. The research show that three *CHS* genes in Asiatic hybrid lily are expressed in anthocyanin-pigmented tepals, but their expression patterns are different (Suzuki et al., 2016).The *CHS2* gene is the key gene involved in bicolor formation of dahlia (Ohno et al., 2018). In this study, three copies of *CHS* gene were cloned, we found that *CHS2* and *CHS3* genes were more closely related than the *CHS1* gene in *I. uliginosa*. And they were expressed in four flower colors, which verified that the *CHS* genes promote anthocyanin synthesis (Cai, 2012; He, 2013). However, the expression of *CHS* genes were the highest in pink flowers, the lowest in white flowers. More importantly, the expression of the three *CHS* genes differed significantly only in pink flowers, and the expression level of *CHS1* gene was the highest. It can also be concluded that the *CHS1* gene played an important role in the anthocyanin biosynthesis in pink flower of *I. uliginosa*.

The basic research of different flower color traits provides support for the further development of flower color breeding, and the diversification of flower color also has a beneficial economic impact on the flower market. In addition, the diversity of flower color can effect pollinator attraction and flower foraging behavior, which has certain ecological significance. Although we have gained some new insights into the influence of internal factors on the four different flower colors, some unexpected results remain to be resolved in the near future. For example, the relationship between anthocyanin content and flavonoids of deep red flowers, and whether pigments with less anthocyanin content were the main pigments for different color differences need to be proved in later experiments.

In general, we suggest that flavonoids of different flower colors in *I. uliginosa* should be detected in future studies. The function of *CHS* gene should also be further verified by transgenic technology, and related transcription factors should be studied to see whether transcription factors have certain regulatory effects on *CHS* gene. In addition, because cellular pH can also affect the formation of different flower colors in *I. uliginosa*, pH-related genes need to be mined and analyzed in the later stage.

## 5 Conclusions

In this study, the internal causes of the formation of four different flower colors in *I. uliginosa* were determined and analyzed. The values of L^*^a^*^b^*^ of petals and the shape of epidermal cells were not the main reasons for the color difference of the four different flower colors, but the cellular pH and anthocyanin and the expression of anthocyanin synthesis *CHS* genes were related to the formation of different flower colors. The results of this study revealed the main internal factors of the formation of different flower colors in *I. uliginosa*, and laid a foundation for the improvement of flower color breeding of *Impatiens*.

## Data availability statement

The original contributions presented in the study are included in the article/**Supplementary Material**. Further inquiries can be directed to the corresponding authors.

## Author contributions

L-QZ: Writing – original draft, Data curation, Formal analysis, Validation. YL: Investigation, Writing – original draft, Data curation, Formal analysis, Validation. QH: Software, Formal analysis, Writing – review & editing. SG: Software, Formal analysis, Writing – review & editing. M-JH: Validation, Writing – review & editing. H-QH: Conceptualization, Writing – review & editing. All authors read and approved the final manuscript.
