# Supplementary material for: Effects of cell morphology, physiology, biochemistry and CHS genes on four flower colors of Impatiens uliginosa
Source: Front Plant Sci. 2024 Mar 1;15:1343830. doi: 10.3389/fpls.2024.1343830 (PMC10940378; doi:10.3389/fpls.2024.1343830)
Supplement: Supplementary file 1 [file DataSheet_1.doc]

**Supplementary Materials:**

Table S1. Information table for acquisition of experimental materials

| Site | Color | [Longitude and Latitude](javascript:;) | Altitude (m) |
| --- | --- | --- | --- |
| Dadie water | Deep Red | 25°08′40″N，102°29′55″E | 1890 |
| Aziying | Red | 25°23′28″N，102°49′34″E | 2113 |
| Laoyu River | Pink | 24°49′18″N，102°46′01″E | 1910 |
| Aziying | White | 25°23′28″N，102°49′34″E | 2113 |

*Table S2. Comparison of pigment species and contents of four kinds of flowers in I. uliginosa*

| **Number** | **Anthocyanin names** | [**Molecular formula**](javascript:;) |
| --- | --- | --- |
| 1 | Cyanidin-3-O-glucoside | C_21_H_21_O_11_ |
| 2 | Cyanidin 3,5-diglucoside chloride | C_27_H_31_O_16_ |
| 3 | Cyanidin-3-O-(2”-galloyl)-β-galactopyranoside | C_28_H_25_O_15_ |
| 4 | Delphinidin | C_15_H_11_ClO_7_ |
| 5 | Delphinidin-3-arabinoside | C_20_H_19_O_11_ |
| 6 | Delphinidin-3,5-diglucoside chloride | C_21_H_31_O_17_ |
| 7 | Malvidin chloride | C_17_H_15_ClO_7_ |
| 8 | Pelargonidin chloride | C_15_H_11_ClO_5_ |
| 9 | Leucopelargonidin | C_15_H_14_ClO_6_ |
| 10 | Peonidin chloride | C_16_H_13_ClO_6_ |
| 11 | Petunidin chloride | C_16_H_13_ClO_7_ |
| 12 | Malvidin-3-O-arabinoside chloride | C_22_H_23_O_11_Cl |
| 13 | Malvidin-3-galactosidechloride | C_23_H_25_ClO_12_ |
| 14 | [Luteolin](http://www.baidu.com/link?url=KkxR15MNoq96Zu89XoyPfcQw6awO0bzutD3fEfE5LYyGimkc9jmL95Ww8eYqPNwd) | C_15_H_10_O_6_ |
| 15 | Kaempferol-3-O-rhamnoside | C_21_H_20_O_10_ |
| 16 | Kaempferol-4'-glucoside | C_21_H_20_O_11_ |
| 17 | Quercetin | C_15_H_10_O_7_ |
| 18 | Quercetin 3-O-xylopyranoside | C_20_H_18_O_11_ |
| 19 | Myricetin | C_15_H_10_O_8_ |
| 20 | Cyanidin-3-O-sambubioside chloride | C_26_H_29_O_15_ |
| 21 | Jaceosidin | C_17_H_14_O_7_ |
| 22 | Peonidin-3-O-galactoside | C_22_H_23_O_17_ |
| 23 | Leucodelphinidin | C_15_H_14_O_8_ |
| 24 | Dihydroquercetin | C_15_H_12_O_7_ |
| 25 | [Tectochrysin](http://www.baidu.com/link?url=n5DYzzT7R95QjvDRhpUMT7nz9taWe_bj_VdPusibmM4TlKG272ykKWWZMXPpJQupZ1The_B1HR-DiyOqWJZosC7oEdMqZA6WH-_YG4km8tW) | C_16_H_12_O_4_ |
| 26 | [Acacetin](https://www.baidu.com/link?url=wJhKyubslWmqqaOgMpm-kNfUZLBvRXgks1TKH52mpF5OyJ3Z5iIJ3CxmBzYAl2nN0IW5AgDjZBDtrCHE6BN7hq&wd=&eqid=e3970bbd000044b10000000664e81576) | C_16_H_12_O_5_ |
| 27 | Quercetin | C_15_H_10_O_7_ |
| 28 | Jaceosidin | C_17_H_14_O_7_ |
| 29 | Quercetin 3-O-rhamnoside | C_21_H_20_O_11_ |
| 30 | Quercetin 3-O-glucoside | C_21_H_20_O_12_ |
| 31 | Myricitrin | C_21_H_20_O_12_ |
| 32 | Irigenin-7-O-beta-D-glucopyranoside | C_24_H_26_O_13_ |
| 33 | [Isorhamnetin 3-O-neohesperidoside](https://www.chemsrc.com/en/cas/55033-90-4_89272.html) | C_28_H_32_O_16_ |
| 34 | Proanthocyanidin A2 | C_30_H_24_O_12_ |
| 35 | B4 Procyanidin B4 | C_30_H_26_O_12_ |

*Table S3. Primers for the full-length cDNA cloning of CHS in I. uliginosa*

| Primer name | Sequence | Purpose |
| --- | --- | --- |
| *CHS*1.AF  *CHS*1.AR  *CHS*2.AF  *CHS*2.AR  *CHS*3.AF  *CHS*3.AR | 5’-ATGGTGACCGTTGAGGAAGTC-3’  5’-ATACCAACACTGTGTAGAACCAC-3’  5’-GTAACCGTCGAGGAAGTCAGG-3’  5’-AACGTCAACACTGTGGAGAAC-3’  5’-GGTCACCGTTCAGGAAGTTAG-3’  5’-TCAAGTACTCACACTGTGAAG-3’ | forward primer  reverse primer  forward primer  reverse primer  forward primer  reverse primer |

Table S4. Primers of target gene of *I. uliginosa* for quantitative real-time PCR

| Primer name | Sequence | Length |
| --- | --- | --- |
| *CHS*1.F  *CHS*1.R  *CHS*2.F  *CHS*2.R  *CHS*3.F  *CHS*3.R | 5’-TGAAGTGGGCTTAACCTTTC-3’  5’-GTTTGAGCTCCACTTGATCC-3’  5’-AGTCTAGTGGGTCAGGCTTTG-3’  5’-GAAACGTCAGACCCACTTCAC-3’  5’-CGGCTGTTATTGTTGGGTC-3’  5’-AATGTAAGGCCCACTTCAC-3’ | 173 bp  172 bp  136 bp |

Table S5. Correlation analysis of *L^*^a^*^b^*^* value of *I. uliginosa*

|  | *L^*^* | *a^*^* | *b^*^* |
| --- | --- | --- | --- |
| *L^*^* | 1 | -0.608 | 0.462 |
| *a^*^* | -0.608 | 1 | -0.905 |
| *b^*^* | 0.462 | -0.905 | 1 |
| N^*^ | 4 | 4 | 4 |

Table S6. Correlation analysis between pH value and *L^*^a^*^b*^*^ value of *I. uliginosa*

|  | L^*^ | a^*^ | b^*^ |
| --- | --- | --- | --- |
| pH值 | -0.946 | 0.352 | -0.149 |
| N | 4 | 4 | 4 |

Table S7. Standard curves for the determination of total flavonoids in *I. uliginosa*

| 标准曲线 | 相关系数（R） |
| --- | --- |
| Abs=0.00344*C+0.01075 | 0.9994 |


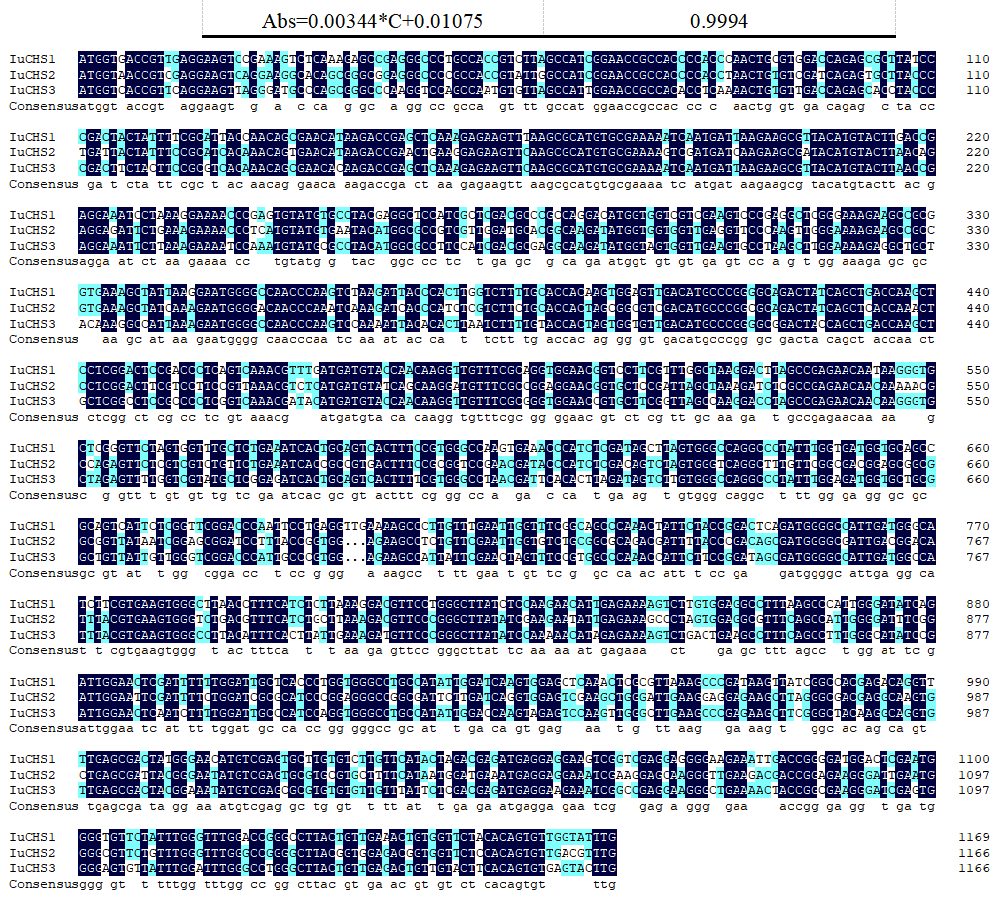


Figure S1. Alignment nucleotide sequences of *CHS* genes of *I. uliginosa*

Table S8. Variance analysis of *CHS1* gene of *I. uliginosa*

|  | DR | R | P | W |
| --- | --- | --- | --- | --- |
| DR | / | 0.694 | 0.002 | 0.026 |
| R | 0.694 | / | 0.005 | 0.000004 |
| P | 0.002 | 0.005 | / | 0.004 |
| W | 0.026 | 0.000004 | 0.004 | / |
| N | 3 | 3 | 3 | 3 |

Table S9. Variance analysis of *CHS2* gene of *I. uliginosa*

Table S10. Variance analysis of *CHS3* gene of *I. uliginosa*

|  | DR | R | P | W |
| --- | --- | --- | --- | --- |
| DR | / | 1.000 | 3.6101E-11 | 7.3047E-9 |
| R | 1.000 | / | 3.3473E-11 | 8.4735E-9 |
| P | 3.6101E-11 | 3.3473E-11 | / | 1.3168E-12 |
| W | 7.3047E-9 | 8.4735E-9 | 1.3168E-12 | / |
| N | 3 | 3 | 3 | 3 |

|  | DR | R | P | W |
| --- | --- | --- | --- | --- |
| DR | / | 0.000003 | 8.5545E-11 | 2.4669E-8 |
| R | 0.000003 | / | 1.2567E-11 | 0.000013 |
| P | 8.5545E-11 | 1.2567E-11 | / | 3.5259E-12 |
| W | 2.4669E-8 | 0.000013 | 3.5259E-12 | / |
| N | 3 | 3 | 3 | 3 |
